# Effects of Imipenem-containing Niosome nanoparticles against high prevalence methicillin-resistant *Staphylococcus Epidermidis* biofilm formed

**DOI:** 10.1038/s41598-022-09195-9

**Published:** 2022-03-24

**Authors:** Tohid Piri-Gharaghie, Neda Jegargoshe-Shirin, Sara Saremi-Nouri, Seyed-hossein Khademhosseini, Eskandar Hoseinnezhad-lazarjani, Aezam Mousavi, Hamidreza Kabiri, Negin Rajaei, Anali Riahi, Ali Farhadi-Biregani, Sadegh Fatehi-Ghahfarokhi

**Affiliations:** 1 Biotechnology Research Center, Microbial Biotechnology Laboratory, AmitisGen Med TECH Group, P.O. Box: 1416673744, Tehran, Iran; 2 Department of Biotechnology, Faculty of Basic Sciences, Damghan Branch, Islamic Azad University, Semnan, Iran; 3 Department of Biology, Faculty of Basic Sciences, Azarbaijan Branch, Azarbaijan Shahid Madani University, Azarbaijan, Iran; 4 Biotechnology Research Center, Islamic Azad University, Tonekabon Branch, Tonekabon, Iran; 5 Biotechnology Research Center, Islamic Azad University, Shahrekord Branch, Shahrekord, Iran; 6 Young Researchers and Elite Club, Shahrekord Branch, Islamic Azad University, Shahrekord, Iran; 7 Sina Borna Aria (SABA) Co., Ltd, Research and Development Center for Biotechnology, Shahrekord, Iran; 8 Department of Biotechnology, Faculty of Basic Sciences, Shahrekord Branch, Islamic Azad University, Shahrekord, Iran

**Keywords:** Biotechnology, Biologics, Nanobiotechnology

## Abstract

We aim to assess the antibacterial and anti-biofilm properties of Niosome-encapsulated Imipenem. After isolating *Staphylococcus epidermidis* isolates and determining their microbial sensitivity, their ability to form biofilms was examined using plate microtiter assay. Various formulations of Niosome-encapsulated Imipenem were prepared using the thin-film hydration method, Minimum Biofilm Inhibitory Concentration (MBIC) and Minimum Inhibitory Concentration (MIC) were determined, and biofilm genes expression was examined. Drug formulations’ toxicity effect on HDF cells were determined using MTT assay. Out of the 162 separated *S. epidermidis*, 106 were resistant to methicillin. 87 *MRSE* isolates were vancomycin-resistant, all of which could form biofilms. The F1 formulation of niosomal Imipenem with a size of 192.3 ± 5.84 and an encapsulation index of 79.36 ± 1.14 was detected, which prevented biofilm growth with a BGI index of 69% and reduced *icaD*, *FnbA*, *EbpS* biofilms’ expression with P ≤ 0.001 in addition to reducing MBIC and MIC by 4–6 times. Interestingly, F1 formulation of niosomal Imipenem indicated cell viability over 90% at all tested concentrations. The results of the present study indicate that Niosome-encapsulated Imipenem reduces the resistance of *MRSE* to antibiotics in addition to increasing its anti-biofilm and antibiotic activity, and could prove useful as a new strategy for drug delivery.

## Introduction

*Staphylococcus Epidermidis*, a species of the coagulase-negative staphylococci (CoNS) family, is the most common bacteria identified on human skin and the most common cause of medical device-related illnesses^1^. The infection largely occurs as the bacteria migrate from the patient’s skin to the surface of the catheter, but they also can migrate via luminal surfaces^2^. This substance, also known as slime or biofilm, allows bacteria to cling to a variety of surfaces^3,4^. Biofilm development includes microbial adherence to and colonization of a surface, cell growth and multilayer creation, maturity, biofilm separation and cell release^5^. The first stage of biofilm formation is adhesion to surfaces, which is facilitated by the bacterium’s *icaD* gene binding. *icaD* (N-acetyl glucosamine transferase) makes up a major part of the exopolysaccharide matrix of the biofilm^6^. Biofilm strength and maturity are then facilitated due to the expression of the *FnbA* and *EbpS* elastin genes^7^. *FnbA* contains multiple fibronectin (Fn) substituents and binding regions, each capable of joining both immobilized and soluble forms of Fn. This enables *S. epidermidis* to attack endothelial cells in vivo and in vitro with no need for additional factors^8^. The elastin-binding protein (*EbpS*) is a 25 kDa cell surface protein encoded by the *EbpS* gene. The binding of *S. epidermidis* to the 30 kDa N-terminal region of elastin, which is the main component of elastic fiber extracellular matrix, leads *S. epidermidis* to colonize the tissue^9^. The most important antibiotics selected for the experimental treatment of infections from *S. epidermidis* (*MRSE*) methicillin-resistant strains are imipenem and vancomycin. Imipenem is a broad-spectrum antibiotic used to treat various types of bacterial infections, including pneumonia, meningitis, sepsis, and anthrax. Imipenem is usually prescribed as a strategic drug to treat patients suffering from systemic infections. According to previous research, using imipenem together with other antibiotics increases their strength against *MRSE* strains^5–9^. However, imipenem overuse over the last decade has resulted in an increased population of enterococci with the imipenem resistance gene. Hence, there is a high probability of plasmids containing resistance genes transferring to *S. epidermidis* strains through conjugation^10^. The bacterium's resistance to antibiotics is due in part to its biofilm structure. This bacterium's methicillin-resistant forms have recently sparked widespread alarm. Because of the increasing frequency of methicillin resistance, hazardous antibiotics such the glycol-peptide vancomycin are now used to treat gram-positive infections. However, intermediate vancomycin resistance was discovered in two strains of *S. epidermidis* and *S. hemolyticus*. This is concerning since there are currently few viable choices for treating staphylococcal infections^11–13^. Researchers are using nanotechnology to produce more novel antibiotics as a result of the rise of bacterial resistance and indeed the slow speed of establishing novel antibiotics in recent decades. Nanomaterials have become one of the best choices for bacterial strain suppression due to their low cost of manufacturing and lack of environmental impact^14^. Encapsulation of antimicrobial agents in nanocarrier systems is one of the most promising and effective approaches to increase antibacterial activity while minimizing adverse effects^15,16^. Niosomes have recently gained popularity as a means of improving selective medication delivery and the therapeutic effectiveness of antifungal agents. Niosomes are bilayer structures with nonionic surfactants that make them water soluble and allow them to transport large amounts of medication^17^. Niosomes contain unique properties that might be utilized to encapsulate a variety of medicines^18^. The usage of Niosomes as antimicrobial nanocarriers has recently piqued researchers' interest^19^. As a result, imipenem was chosen as a powerful antibiotic against *MRSE* strains in the current investigation to minimize antibiotic resistance and boost antimicrobial effects. The goal of this research was to create Niosome-encapsulated imipenem with enhanced antibacterial activity against *MRSE* strains.

## Materials and methods

### Ethics approval and consent to participate

The authors of this article state that all methods are reported in accordance with ARRIVE guidelines (https://arriveguidelines.org). All protocols were performed in accordance with the Ethical Committee and Research Deputy of the Islamic Azad University of Shahrekord Branch, Iran for the Care and Use of Laboratory Animals and were approved by the Institutional Animal Care and Use Committee guidelines of Islamic Azad University, Shahrekord, Iran (IR.IAU.SHK.REC.1400.043). Ethics approval needed for microbial testing and isolation of pathogenic bacteria in Iran.

### Preparation of Niosomes encapsulated Imipenem

#### Formulation and encapsulation of Imipenem in noisome

According to instructions provided by previous studies^20,21^, Noisome encapsulation of imipenem was conducted through thin-film hydration, and 2 drug compounds were prepared as follows. The first compounds were prepared. The first compound contained Span 60 (CAS: 1338-41-6) (Henan Daken Chemical CO., LTD., China) and Tween 60 (CAS: 9005-67-8) (Career Henan Chemical Co, China) mixed with cholesterol (CAS: 57-88-5) (Capot Chemical Co., Ltd., China) at molar ratios of 3:3:4 and was dissolved in 10 ml of chloroform and methanol mixed at ratios of 2:1. The second compound contained Span 40 (CAS: 26266-57-9) (Henan Daken Chemical CO., LTD., China) and Tween 40 (CAS: 9005-66-7) (Shanghai Jizhi Biochemical Technology Co., Ltd, China) mixed with cholesterol (CAS: 57-88-5) (Capot Chemical Co., Ltd., China) with respective molar ratios of 3:3:4 and was dissolved in 10 ml of chloroform and methanol mixed with respective ratios of 2:1 (Table 1). After adding glass beads to all 2 compounds, drug compound solvents were evaporated using a rotary evaporator (Heidolph, Germany) for one hour at 60 °C and 120 rpm rotation. Afterward, dried thin films were hydrated for one hour using a solution of imipenem dissolved in 10 ml of PBS at 60 °C with a speed of 120 rpm to obtain different Niosome formulations. The minimum inhibitory concentration of imipenem against Staphylococcus (1 mg/ml) was used as the concentration of this drug for encapsulation. The resulting particles were sonicated using a probe sonicator in an ice bath using SONOPULS ultrasonic homogenizers (amplitude: 25%, 200 wt) for five minutes to reduce the size of Niosomes containing imipenem, and samples were stored at 4 °C for subsequent experiments.

**Table 1:** Preparation of various Niosome-encapsulated imipenem formulations.

Formulations	Different compounds of surfactant:cholesterol	Span + tween + cholesterol molar ratio	Span:tween molar ratio	Drug concentration (mg/ml)
**Different formulations of Niosomal Imipenem**				
F1	Span 60 + Tween 60 + Cholesterol	3:3:4	50:50	1
			25:75	1
			75:25	1
F2	Span 40 + Tween 40 + Cholesterol	3:3:4	50:50	1
			25:75	1
			75:25	1

#### Noisome-encapsulated imipenem morphological characteristics

The average size, polydispersity index, and zeta potential of imipenem loaded in noisome were obtained using dynamic light scattering (DLS) and ZetaPlas palladium electrodes (Brookhaven Instruments Corp., USA). The polydispersity index (PDI) is a size-based metric for assessing sample variability. Polydispersity can develop as a result of a pattern's particles size or accumulation or aggregation during extraction or evaluation. PDI was measured by DLS (Brookhaven Instruments Corp., USA). Various newly prepared Niosome formulations were diluted two times using distilled water at a ratio of 20:1 to prevent multiple scattering as a result of interactions between particles, and analysis was conducted at 25 °C with a 90-degree light scattering angle. The average z diameter and Niosome multiple scattering index were determined, and their zeta potentials were measured. Niosome-encapsulated imipenem particles were coated with a gold layer to generate electrical conductivity and were examined using an electron microscope (SEM) device model MIRA3 (TESCAN, Czech Republic).

#### Entrapment efficiency (EE)

Encapsulation efficiency was obtained by determining the amount of noncapsulate imipenem (free imipenem) in the formed Niosomes. Noisome-encapsulated imipenem particles were isolated over an hour at 4 °C in a refrigerated centrifuge at 1400 rpm. The imipenem content of the supernatant was examined through ELISA Reader Stat Fax2100’s (Awareness Technology, Ukraine) light absorbance reading of the supernatant at 276 nm wavelength, and the EE percentage was obtained based on Formula (1).

1 $$ \% {\text{EE }} = \, \left( {\left( {{\text{Drug added }}{-}{\text{ Free }}\hbox{``}{\text{unentrapped drug}}\hbox{''}} \right)/{\text{Drug added}}} \right) \, \times { 1}00 $$

#### Evaluation of noisome-encapsulated release and stability

Dialysis was employed to examine imipenem’s release of the noisome. The dialysis tube was soaked in distilled water for 24 h. Then, 0.5 ml (10 mg) of imipenem-loaded noisome was placed in a dialysis bag, and 0.5 ml imipenem antibiotic aqueous solution containing 10 mg imipenem was also used as a control sample. Dialysis bags were immersed in conical flasks in 75 ml distilled water and shaken at 50 rpm in a water bath at 37 °C. Five milliliters were withdrawn from the receptor medium at intervals of one, two, four, six, 12, and 24 h, Aliquots of samples were replaced with a new medium at 37 °C, and imipenem was measured by spectrophotometry at 281 nm. The diffusion profile was determined using various kinetic models. This method was used to monitor the stability of diffusion of various Niosome-encapsulated imipenem formulations at intervals of 7, 14, 21, 28, 35, 42, 49, and 56 days for a storage period of two months at 25 °C.

### Investigation of antibacterial activity

#### Bacterial isolation and antibiotic susceptibility

This descriptive study collected a total of 300 clinical samples from Shariati (150 samples), Firoozgar (150 samples), in Tehran. The samples used are from hospitals bio-bank. Samples were cultivated in blood agar cultures using sterile swabs at 37 °C for 24 h. First, *S. epidermidis* isolates were detected using Gram staining, catalase, mannitol fermentation, and DNase tests. Then identification of the species was performed by analyzing the 16S rRNA gene. Strain sensitivity to various antibiotics was examined by disc diffusion according to the Clinical and Laboratory Standards Institute (CLSI, CLSI supplement M100 (ISBN 1-56238-804-5 (Print); ISBN 1-56238-805-3 (Electronic)) method after *S. epidermidis* strains were detected and confirmed. *S. epidermidis* isolates’ sensitivity to vancomycin (VA, 10 μg), cefoxitin (FOX, 10 μg), ciprofloxacin (CIP, 5 μg), clindamycin (CD, 2 μg), mupirocin (MU, 5 μg) and imipenem (IMI, 10 μg) (MAST, UK) discs was measured in Muller Hinton agar culture (Merck, Germany). It must be noted that cefoxitin antibiotic discs were used to detect *MRSE* methicillin resistance, and the *S. epidermidis* ATCC 14990 standard strain was used as the positive control. Multidrug-resistant (MDR) was defined as acquired non-susceptibility to at least one agent in three or more antimicrobial categories.

#### DNA extraction process

The bacterial genomic DNA was extracted according to the directions of the Cinna Gene company's extraction kit (Cinna Pure DNA KIT, Alborz, Iran). Briefly, the bacterial solution was centrifuged. After centrifuging the bacterium, the precipitate was mixed with 100 μl of proteinase K and protease buffer and heated at 55 °C for 30 min. Lysis buffer was introduced in 200 μl and vortexed for 15 s. Afterward, 300 μl of precipitation solution was added and inverted ten times before being subjected to – 20 °C for one hour. The supernatant was drained after 10-min centrifugation at 13,000 rpm, then 700 μl of wash buffer was added and centrifuged for 5 min at 13,000 rpm. The PCR test was used to identify 16S rRNA gene.

#### Molecular detection of *icaD*, *FnbA*, and *EbpS *biofilm genes and resistance to vancomycin *VanB*

The bacterial genomic DNA was extracted according to the directions of the Cinna Gene company's extraction kit (Cinna Pure DNA KIT, Alborz, Iran), and the purity was verified using a spectrophotometer at 260 nm. The M-PCR test was used to identify biofilm decoding genes *icaD*, *FnbA*, *EbpS* and the vancomycin resistance gene *VanB* utilizing oligonucleotide sequences of particular primers listed in Table 2. Primer design for genes *icaD* (AAQ88121.1), *FnbA* (KAB2208440.1), *EbpS* (WP_032604310.1) and *VanB* (OLS07309.1) were performed with Oligo 7 software and the results were reviewed in the online blast tool in the NCBI database. The final reaction volume (50 µL) was considered to contain 30 µL PCR master mix (PCR buffer, MgCl2, dNTP, 0.2 units of Taq polymerase), 0.5 µL *icaD* reverse primer, 0.5 µL *icaD* forward primer, 0.5 µL *FnbA* reverse primer, 0.5 µL *FnbA* forward primer, 0.5 µL *EbpS* reverse primer, 0.5 µL *EbpS* forward primer, 2 µL template DNA, and 15 µL distilled water (Amplicon, Denmark). The PCR program was performed in the form of an initial denaturation at 95 °C for five minutes and 35 initial denaturation cycles for one minute at 94 °C, one minute of annealing at 58 °C, one minute of extension at 72 °C, and a final extension at 72 °C for five minutes. The amplified products were investigated through 1% agarose gel electrophoresis to detect the desired genes of *vancomycin resistance*. *Enterococcus faecalis ATCC 51299* were used as Vancomycin-resistant control.

**Table 2:** Sequence of primers used in this research.

Genes		Primer sequence 5ʹ → 3ʹ
**Primers used for screening**		
Reference gene	16SrRNA	F: TACATGCAAGTCGAGCGAAC R: AATCATTTGTCCCACCTTCG
Biofilm genes	*icaD*	F: ACCCAACGCTAAAATCATCG R: GCGAAAATGCCCATAGTTTC
	*FnbA*	F: AAATTGGGAGCAGCATCAGT R: GCAGCTGAATTCCCATTTTC
	*EbpS*	F: GGTGCAGCTGGTGCAATGGGTGT R: GCTGCGCCTCCAGCCAAACCT
Vancomycin resistance gene	*VanB*	F: GTG ACA AAC CGG AGG CGA GGA R: CCG CCA TCC TCC TGC AAA AAA

#### Antibacterial activity of noisome-encapsulated imipenem

The antibacterial activity of various noisome-encapsulated imipenem formulations against *MRSE* strains was examined through broth microdilution. The minimum inhibitory concentration (MIC) and minimum bactericidal concentration (MBC) were then determined as follows. The MIC is the lowest concentration of a chemical, usually a drug, which prevents visible growth of a bacterium. The MBC is measured by sub-culturing the broths used for MIC determination onto fresh agar plates. MBC is the lowest concentration of a drug that results in killing 99.9% of the bacteria being tested. To determine the MBC, the dilution representing the MIC and at least two of the more concentrated test product dilutions are plated and enumerated to determine viable CFU/ml. The closer the MIC is to the MBC, the more bactericidal the compound. After culturing *S. epidermidis* in Müller-Hinton broth for 24 h, 5 × 10^5^ CFU/mL (5 μl) bacteria, 100 μl of various Niosome-encapsulated imipenem formulations, free imipenem and free Niosome (at a concentration ranging from 0.03 to 64 μg/ml), and 95 μl pure Hinton broth were poured into each well of a 96-well plate and incubated for 24 h at 37 °C. The sub-MIC values of negative control well (containing pure culture medium), and positive control well (containing culture medium plus standard *S. epidermidis* strain ATCC 14,990) were determined, and the test was replicated for a 3 time.

#### Inhibition of biofilm formation

Sub-MIC concentrations of free imipenem and Niosomes and various formulations of Niosome-encapsulated imipenem were used to determine the minimum biofilm inhibition concentration (MBIC) against the *S. epidermidis* strains. The wells of the 96-well plate were filled with 100 μl drug sample and 100 μl of the bacteria cultured in Müller Hinton Broth. The plates were incubated for 48 h at 37 °C and stained with crystal violet 1% after being washed. The tests were replicated two times, and the mean MBIC was determined to be OD630 < 0.1.

#### Analysis of biofilm gene expression

The expression of the biofilm genes *icaD*, *FnbA*, and *EbpS* was examined through polymerase chain reaction (qRT–PCR) using the specific primers indicated in Table 2. RNA was extracted from resistant *MRSE* bacteria using an RNX-Plus kit (Sina gene, Iran) after 24 h of exposure to sub-MIC concentrations of free imipenem and Niosome-encapsulated imipenem, and cDNA was fabricated based on the RNA extracted from treated and untreated bacteria according to the protocol of the YTA Kit (Yekta Tajhiz, Iran). Biofilm gene expression to the 16S rRNA gene (as the reference gene) was examined using a Corbett 5 Plex HRM real-time PCR device (Corbett, Australia). The final reaction volume was 15 μl, containing 1 μl of cDNA, 1 μl of the forward primer and 1 μl of reverse primer, 8 μl of master mix and 4 μl of deionized water (Merck, Germany), and the temperature cycle included initial denaturation for five minutes at 95 °C followed by 40 cycles of 20 s at 95 °C, 40 s at 58 °C, and 40 s at 72 °C. The final stage was selected to be at 53–95 °C to draw melting curves. Gene quantitative relative expression was examined using the ΔΔCt method.

#### Cytotoxicity study

According to standard ISO10993-5 and Li et al.^22^, the cytotoxicity test was performed by the MTT method on HDF normal cells. To check the cytotoxicity, an L929 confluence flask was placed under a hood, and the cells were cultured in fully sterile DMEM with high Glu (catalog number 11965118) (Thermo Fisher Scientific, United States) culture medium^23^. The surface of the cells was washed with PBS, and trypsin was added to remove the cells from the fibroblastic state and make them spherical. Then, DMEM containing 10% FBS (bovine fetal serum) was poured onto the cells to neutralize trypsin, and the cells were poured into a Falcon tube and centrifuged at 1200 rpm for 5 min. The supernatant was discarded, DMEM containing 10% FBS was poured, and cell counts were performed on a Neobar slide. For each well, a 24-cell plate of 5 × 10^4^ cells were considered. Sterile samples of free imipenem and niosomal encapsulated imipenem were also placed in the center of each plate under sterile conditions, and the cell suspension was added and placed in a 37 °C incubator containing 5% CO_2_ for 24 h. According to standard ISO10993-5, after 24 h, the medium was removed, the cells were washed with PBS, and then a sufficient amount of MTT (1 mg/ml) was poured onto the cell layer. The culture plate was incubated for 3 to 5 h, and the cells were then washed with PBS to remove unreacted MTT. The product of Formosan solvent by isopropanol and OD560 nm of each cell was evaluated. Cell viability was obtained from the absorption fraction of each sample on the control sample (polystyrene container). Additionally, the percentage of living cells or the rate of cell survival was calculated based on the following Eq. (2):

2 $$ {\text{Viable cell percentage}}\% \, = {\text{ treated cells mean absorbance}}/{\text{control cells mean absorbance}} \times {1}00 $$

### Statistical analysis

Statistical analysis in this study was calculated using SPSS software version 16, and the results were subjected to one-way analysis of variance (ANOVA). Additionally, the expression of target genes between the control and treated samples was calculated by Tukey’s HSD post statistical method. The confidence interval (CI) of the analysis was 95% (CI 2.23–4.23, P < 0.001).

### Ethics approval and consent to participate

The study was approved by the Committee of Islamic Azad University of Shahrekord Branch in Iran (IR.IAU.SHK.REC.1400.043).

## Results

### Imipenem encapsulated in Niosomes has a uniform and spherical structure with suitable morphological features

Various formulations of Niosome-encapsulated imipenem were studied morphologically and in terms of various indicators, such as the surfactant-to-cholesterol ratio, Span-to-Tween ratio, and the composition of different surfactants Span and Tween (Table 1). Each of the formulations had a distinct size, polydispersity index (PDI), and entrapment efficiency (EE). Dynamic light scattering (DLS) revealed that various formulations of Niosome-encapsulated imipenem had good uniformity (Table 3). As demonstrated, F1 is of a smaller and better size and is associated with surfactant Span60’s hydrophile-lipophile balance. Span60 has a hydrophile-lipophile balance of 4.7, while the corresponding value in Span40 is 6.7. Therefore, nanoparticles formulated with Span60 are smaller. In addition, the EE content of the F1 formulation was higher than that of the other formulations, which might be due to the surfactant used. A longer saturated alkyl chain is directly associated with the permeability of drugs in Niosomes so that longer saturated alkyl chains will result in greater permeability. Span60 has a longer alkyl chain than Span40, which is why formulations incorporating Span60 have higher indexes and EE content is at its peak in the F1 formulation. Polydispersity indexes (PDI) smaller than 0.3 indicate a suitable distribution of small nanoparticles, indicating that the F1 formulation is the optimal formulation given that it has the smallest PDI (Table 3).

**Table 3:** Morphological features of Niosome-encapsulated imipenem compared to blank Niosomes.

Formulations	Polydispersity index (average ± SD)	Zeta potential (m.v)	Vesicle size (nm) (SEM)	EE (%)
Blank Niosome	0.307 ± 0.69	− 73.22 ± 1.05	0.601.22 ± 0.14	–
F1	0.183 ± 0.02	− 65.29 ± 2.68	192.3 ± 5.84	79.36 ± 1.14
F2	0.281 ± 0.013	− 68.75 ± 2.84	218.1 ± 14.26	61.12 ± 1.03

**Figure 1:**
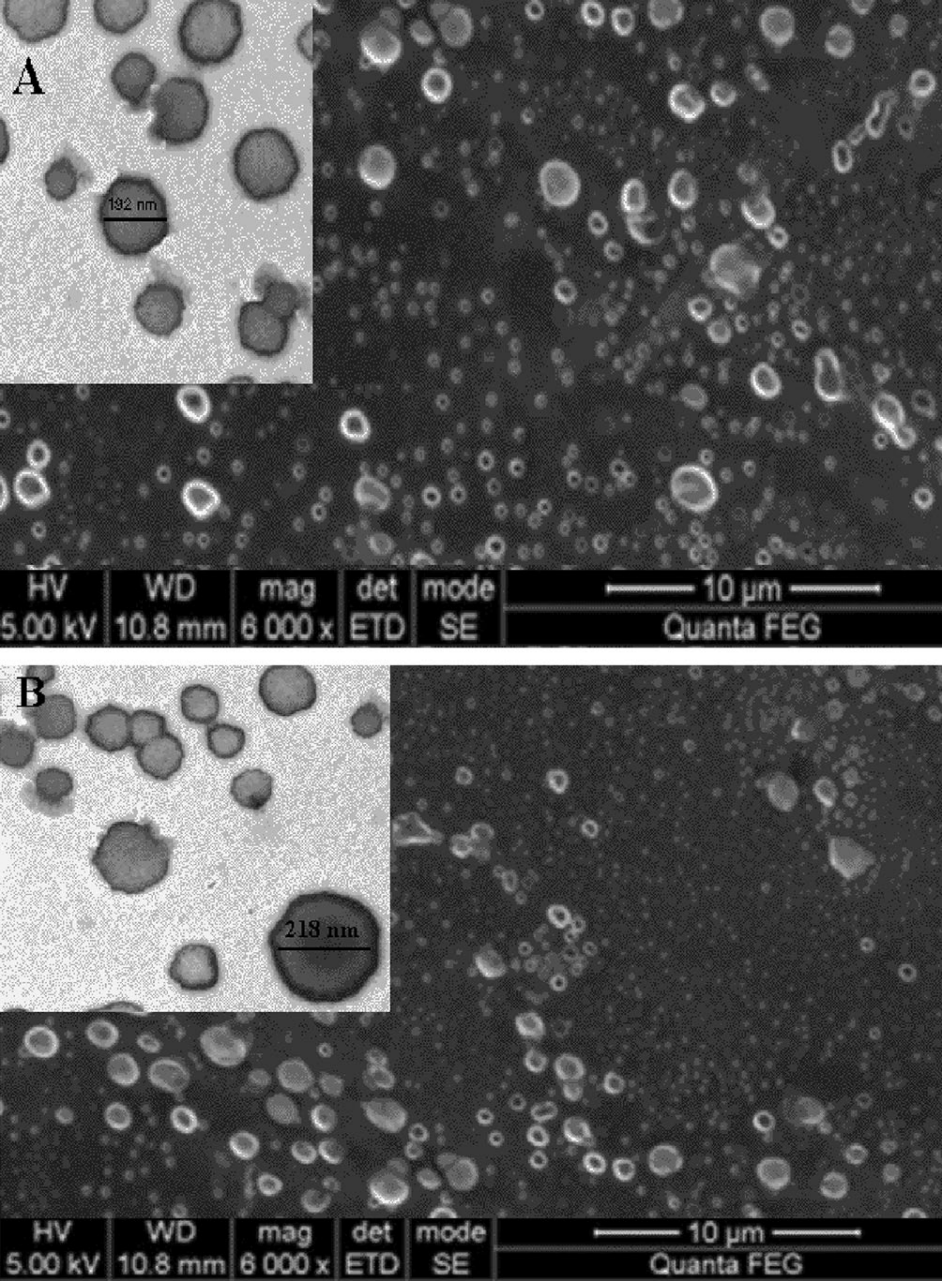
(**A**) F1 formulations of Niosome-encapsulated Imipenem. (**B**) F2 formulations of Niosome-encapsulated Imipenem.

As Fig. 1 demonstrates, SEM results indicated a uniform spherical shape in the Niosome-encapsulated imipenem of the F1 formulation with an average size of 192.3, which indicates the suitable diameter (< 200) of this drug formulation.

**Figure 2:**
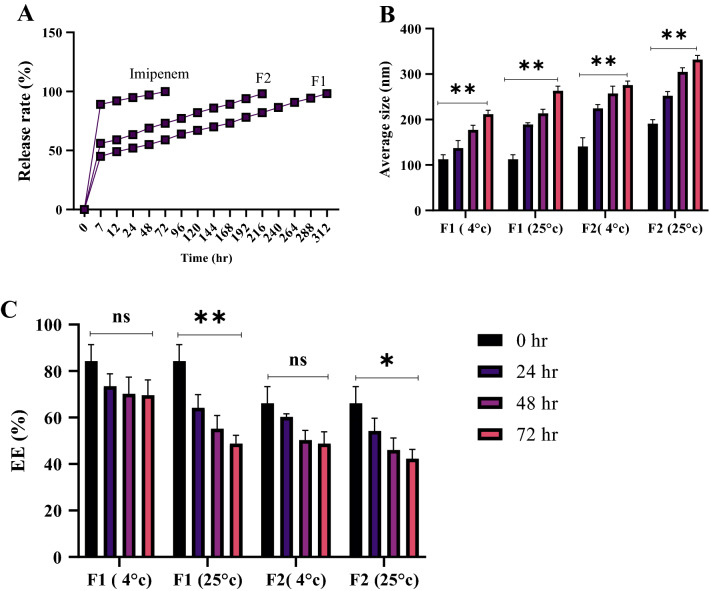
(**A**) The rate of controlled drug release in various formulations of Niosome-encapsulated Imipenem compared to free Imipenem. (**B**) the impact of temperature on average size and encapsulation efficiency (**C**) of F1 and F2 formulation of Niosome-encapsulated Imipenem (ns, *p* > 0.05, ** *p* < 0.01, * *p* < 0.05. n = 3).

The amount of imipenem released from the Niosome was examined using the dialysis bag method. Imipenem’s antibiotic release from Niosome nanoparticles demonstrated a two-phase release pattern made up of the blast and stable phases so that the stable constant reached its peak after 7 h in various formulations. The released amount of antibiotic then started declining, and the release speed increased with a gentle slope for up to 72 h. Lower speeds in drug release in the blast phase were associated with higher encapsulation efficiency and more effective controlled drug release. Therefore, the highest efficiencies of controlled emission were attributed to formulations F1 and F2 with respective release percentages of 45 and 56 in the blast phase. The F1 formulation had the highest efficiency in controlled diffusion, showing 98.2% drug release after 312 h, while free imipenem drug release was 89% in the blast phase, which increased to 99% in 72 h (Fig. 2A).

The morphological features of Niosome-encapsulated imipenem, such as the average size and encapsulation efficiency (EE), were examined to evaluate its stability at various temperatures and time intervals. The results showed that increasing the temperature affects the morphological aspects of Niosome-encapsulated imipenem and increases the size, resulting in a decrease in drug EE, but extending the life span had no significant influence on its EE or morphological features. (Fig. 2B,C). According to the results, various drug formulations had higher stability at 4 °C than at 25 °C, indicating the negative impact of temperature on encapsulated drug stability. On the other hand, the F1 formulation of Niosome-encapsulated imipenem was the most stable among the formulas.

### The high frequency of *MRSE* strains is found in Iran Hospitals

Out of the 300 collected samples, 162 cases were confirmed to contain *S. epidermidis* after microbial diagnostic tests, and further molecular examination by 16S rRNA gene confirmed the identification of *S. epidermidis* strains. Shariati Hospital had the highest frequency of 87 patients (53.7%), and Firuzgar Hospital had the lowest frequency of 75 patients (46.29%), ranking first to last in terms of *S. epidermidis* infection considering the number of samples taken from the hospitals under study. Afterward, the sensitivity of *S. epidermidis* isolates to various antibiotics was studied (Fig. S1), which indicated that out of the 162 studied samples, 134 isolates (82.71%) were multi-drug resistant (MDR), while 106 isolates (65.43%) were methicillin-resistant (*MRSE*). In addition, 97 samples (72.38%) out of the 134 MDR isolates were capable of forming biofilms (Table 4).

**Table 4:** Screening and determining the frequency of *MRSE* and MDR in biofilms from various hospitals.

Biofilm production		Biofilm genes			
Total	Biofilm phenotype	Number	*icaD*	*FnbA*	EbpS
Shariati Hospital (64)	Strong	31	+	+	+
	moderate	19	+	+	+
	Weak	11	−	+	+
	Not connection	3	—	—	—
Firuzgar Hospital (33)	Strong	18	+	+	+
	moderate	5	+	+	+
	Weak	8	+	−	−
	Not connection	2	—	—	—
97	Weak sum = 19	Moderate sum = 24		Strong sum = 49	
Measurement criteria	Strong Biofilm: OD > 4 × ODc moderate Biofilm: 2 × ODc ˂ OD ≤ 4 × ODc Weak Biofilm: ODc ˂ OD ≤ 2 × ODc Not connection: OD ≤ ODc ODc = 0.0025				

### Imipenem encapsulated in Niosomes has strong antibacterial and antibiofilm properties

Two drugs of free imipenem and various compositions of Niosome-encapsulated imipenem were evaluated against MDR *S. epidermidis* (that were resistant to vancomycin and methicillin) to analyze the antimicrobial features of MIC, MBC, and sub-MIC Niosome-encapsulated imipenem, and the results are listed in Table 5. MIC values for free imipenem range between 4 and 8 μg/ml, to which the aforementioned strains indicated various degrees of resistance. In addition, MBC values did not indicate significant differences from MIC values. The antibiofilm activity of Niosome-encapsulated imipenem compared to free imipenem was also examined through MBIC. However, among all the examined samples, the F1 formulation decreased the amount of Niosome-encapsulated imipenem by 4–6 times the MBIC in comparison with free imipenem. Other formulations of Niosome-encapsulated imipenem (F2) indicated other MIC, MBC, and MBIC degrees. On the other hand, free imipenem had no lethal effect or growth inhibition at any of the aforementioned concentrations.

**Table 5:** Values of MIC, MBC, and MBIC in various Niosome-encapsulated imipenem formulations and free imipenem against resistant *S. epidermidis* strains.

Bacterial strain	Sample/isolation site	Pathogenic factors				Free Imipenem			Encapsulated Imipenem			
		Biofilm Phenotype	MRSE	MDR	*VanB* Resistance Gene	MIC (μg/ml)	MBC (μg/ml)	MBIC (μg/ml)	Formulation	MIC (μg/ml)	MBC (μg/ml)	MBIC (μg/ml)
SMRSE1	Blood/Shariati Hospital	Strong	+	+	+	4	8	2	F1	0.25	0.50	0.125
									F2	1.0	2.0	0.50
SMRSE2	Blood/Shariati Hospital	Strong	+	+	+	4	4	2	F1	0.125	0.25	0.062
									F2	0.50	1.0	0.25
SMRSE3	Blood/Shariati Hospital	Strong	+	+	+	8	16	4	F1	0.25	0.50	0.125
									F2	2.0	4	1.0
SMRSE4	Blood/Firuzgar Hospital	Strong	+	+	+	2	4	2	F1	0.125	0.25	0.125
									F2	1.0	1.0	0.50

**Figure 3:**
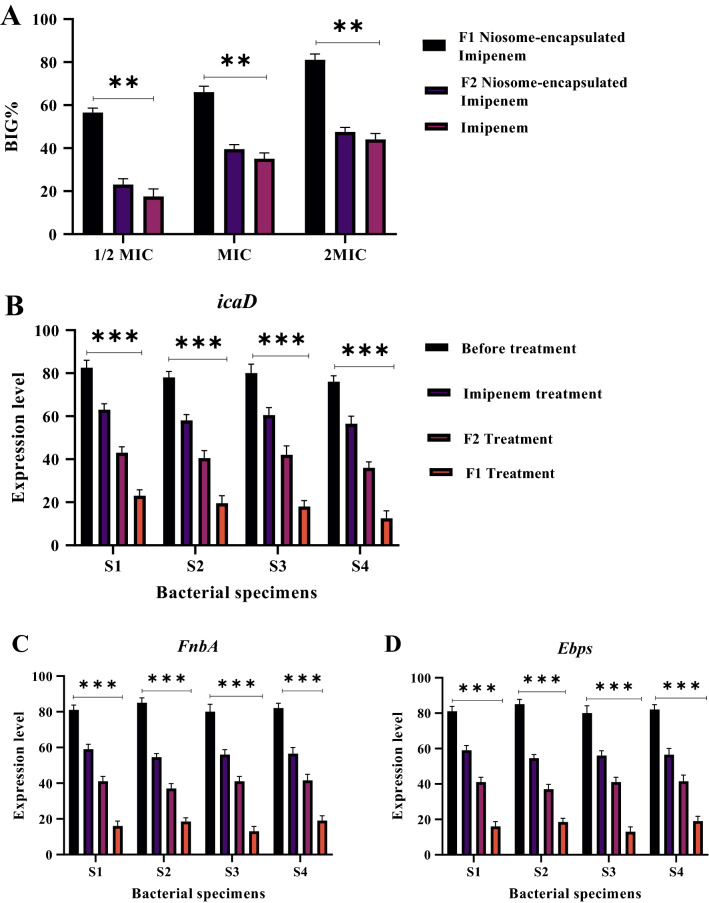
(**A**) Biofilm growth inhibition percentage of various formulations of Niosome-encapsulated Imipenem. (**B**–**D**) Effect of various drug formulations on biofilm gene expressions. The results show that F1 formulation has the greatest effect on reducing the expression of biofilm genes (ns, *p* > 0.05, *** P < *0.001*, ** *p* < 0.01, * *p* < 0.05. n = 3).

Out of the examined bacterial strains, the 4 isolates S*MRSE*1, S*MRSE*2, S*MRSE*3, and S*MRSE*4 were selected as biofilm-generating strains, and the ability of Niosomes carrying imipenem to inhibit the growth of resistant *S. epidermidis* isolates over a short period of exposure to drug formulations was compared to that of free imipenem. Compared to the minimum biofilm inhibitory concentration (MBIC) study, biofilm growth inhibition conditions were harder since biofilms were first treated for only two hours with drug formulations or free imipenem; biofilms were then washed and incubated in antibiotic-free medium for 24 h. This study was conducted at concentrations ranging from 1/2 MIC to 2MIC for the respective isolates. The concentration of drug formulations compared to free imipenem MIC was calculated for physical composition, and the results were reported as the biofilm growth inhibition percentage (BIG%, Fig. 3A).

Afterward, subMIC concentrations of Niosome-encapsulated imipenem were used to examine antibiofilm activity, and the expression of biofilm genes *icaD*, *FnbA*, and *EbpS* in bacteria was examined through quantitative real-time PCR. The RNA concentration was previously normalized. The results obtained from gene expression to the 16S rRNA reference gene in positive biofilm strains were recorded and are shown in Fig. 3B–D. According to the results, the highest decline in the expression of the *icaD*, *FnbA* and *EbpS* genes was observed after treatment with Niosome-encapsulated imipenem (P ≤ 0.05). In addition, the F1 formulation had the highest efficiency among the drug formulations.

### Niosome-encapsulated imipenem has the lowest cytotoxicity against the normal HDF cell line

**Figure 4:**
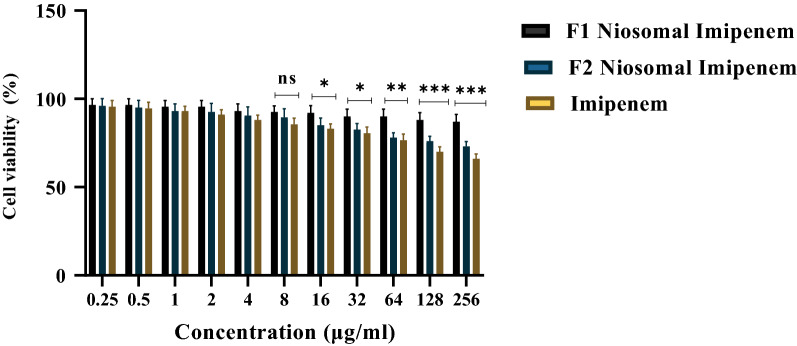
Cell viability percentage of HDF cells treated with various formulations of Niosome-encapsulated Imipenem in comparison with free Imipenem over 24 h (ns, *p* > 0.05, *** *p* < 0.001, ** *p* < 0.01, * *p* < 0.05. n = 3).

Eventually, the cytotoxicity of various drug formulations was compared to that of free imipenem on the HDF cell line. Niosome encapsulation indicated a considerably higher cell viability rate than free imipenem at all tested concentrations. Almost 68% of the cells survived for 24 h after being treated with 256 μM imipenem, while over 90% of cells survived for 24 h after being incubated with the F1 formulation of Niosome-encapsulated imipenem. Cell viability was 96%, 85% and 74% for the respective formulations of F1, F2, and Free Imipenem at 256 μM (Fig. 4).

## Discussion

Methicillin-resistant *S. epidermidis* is a prevalent hospital pathogen whose prevalence has increased worldwide over the past three decades and has posed serious threats as a nosocomial infection^24^. Hence, examining this pathogen's prevalence in samples taken from 2 hospitals, Shariati, Firouzgar, indicated a 54% *S. epidermidis* prevalence. On the other hand, *S. epidermidis* (*MRSE*) bacteria demonstrate resistance to a variety of antibiotics, and some of its strains have indicated vancomycin resistance^25,26^. The present study examined the sensitivity of various isolates, indicating that 82.71 of the isolates were MDR, which was consistent with previous research^27^. In addition, 72.38% of the isolates were capable of forming biofilms, which are among the main factors of microbial resistance among Iranian nosocomial infections according to Piri et al.^28^. According to the literature, the formation of biofilms in *S. epidermidis* reduces the speed of antibiotic penetration and causes complications for the treatment of infections due to these bacteria^29^. Therefore, research focused on new treatment strategies as well as strategies for reducing *MRSE* resistance is of great importance. Given that Niosome formulations are being studied as promising tools to increase compounds' antibacterial activities^30^, the present study focused on Niosome-encapsulated imipenem to reduce the resistance of *MRSE* and increase antibacterial activity against *S. epidermidis*. Various synthesized formulations were prepared, including Tween 60 and Tween 40 nonionic surfactants as well as Span 60 and Span 40 mixed with cholesterol. Given that various cholesterol levels could result in various bilayers in formulations and impact bilayers’ surface load, its amount was kept steady across all formulations. Therefore, the Niosome membrane's strength and resistance to ultrasound waves were kept constant across all formulations by keeping the cholesterol constant, which resulted in more uniform vesicles in terms of size^31^. On the other hand, various surfactant combinations resulted in nanostructures with various morphological features^32^. The use of equal Tween and Span ratios resulted in different results. Tween 60 results in reduced Niosome membrane strength because of its high hydrophilicity^33^. This defect was resolved by adding an equal Span 60 surfactant (50:50), which is more hydrophobic and results in the formation of dense Niosome films. On the other hand, Tween 60 and Span 60 have higher phase transfer temperatures than the other surfactants used in the study, which results in increased drug encapsulation^34^. Span60’s long saturated alkyl chain resulted in more preamble Niosomes as an influential factor, resulting in multiplied drug encapsulation^35^. In addition, the Span60 surfactant has a lipophilic balance of 4.7, which is lower than that of Span40 (6.7), resulting in the formation of smaller vesicles^36^. Therefore, the F1 formulation was revealed to have a more suitable size and morphological features than the F2 formulations. The Span40 used in F2 formulations resulted in larger vesicles compared to that of F1 due to their high hydrophilic-lipophilic balance. On the other hand, formulation F2 contained fewer encapsulated drugs despite their larger size due to Span40's unsaturated alkyl chain, resulting in their lower encapsulation efficiency (EE). In addition, Formulation F2 had lower phase transfer temperatures and weaker wall strength due to the presence of Tween 60 and Tween 40 surfactants, which resulted in the formation of a thin structure and low encapsulation efficiency. Hence, the size obtained for the F1 formulation was quite smaller than the other formulations, indicating this formulation's higher EE. Hedayati et al. examined various Niosome-encapsulated drug formulations, reporting that the size of Niosome-encapsulated drugs varied across different formulations^37^. This study’s-controlled drug release profile indicates a biphasic pattern. Given the Niosome bilayer structure, the drug might reside either in the center of the Niosome within the two layers or at the surface of the Niosome during encapsulation^38^. Therefore, the drugs on the surface start being released over the first hours of release, which results in the blast phase (fast drug release). After seven hours, surface drugs will have released, and encapsulated drugs residing at the center of the Niosome and on the bilayer membrane start being released, resulting in controlled drug release and the stable phase. On the other hand, the cholesterol present in the system stops the gel-to-lipid phase transfer in Niosome systems, which prevents the drug from leaking out of the Niosomes, resulting in controlled drug release over the long term and longer efficient drug release^39^. Because of its higher strength and good surface density, formulation F1 forms better links with cholesterol, resulting in a reduced drug delivery process. In addition, negative zeta potentials are due to the electrostatic repulsion between the particles according to previous research, resulting in higher Niosome stability^40^. Studying the zeta potential and stability revealed that formulation F1 had the most stable Niosome-encapsulated imipenem. Additionally, the drug stability was higher at 4 °C than at 25 °C, which might be due to the lower Niosome bilayer mobility at 4 °C^41^. As a negative factor, the time of drug immobility has a direct relationship with nanoparticle size and an inverse relationship with the EE index. A longer drug immobility time results in a larger nanoparticle size due to particle accumulation or fusion, and the pressure resulting from nanoparticle accumulation on the Niosome bilayer might result in the diffusion of the layers and a reduction in their EE^42^. The present study mainly aimed to improve the antibacterial features of Niosome-encapsulated imipenem. Niosome-encapsulated imipenem had a much lower MIC than free imipenem, indicating the drug's increased efficiency and antibacterial activity and bacteria's lower drug resistance. Barakat et al. suggested that loading vancomycin in the Niosome improves its antibiofilm and antibacterial activity and efficiency against Staphylococcus^40^. Numerous studies have reported Niosomes have the potential to increase antibiotic antibacterial activity through targeted drug delivery and protect antibiotics from unfavorable environmental conditions^43^. Kopermsub et al. reported the great potential of Niosomes to encapsulate niacin and improve their antibacterial activity^44^. Theansungnoen et al. reported that the cholesterol existing in Niosomes results in increased targeted drug delivery to bacteria through binding to bacterial lipid membranes^45^. Consistent with that of Machado and Akbari, the results of the present study indicate increased antibacterial activity in nanomaterial-encapsulated antibiotics^46,47^. According to Gupta et al., there are various absorption and fusion mechanisms between the Niosome and the bacterial wall that could result in increased targeted drug delivery^48^. The other aim of the present study was to examine Niosome-encapsulated imipenem’s antibiofilm features in the form of biofilm formation inhibition, biofilm growth inhibition, and prevention or reduction of biofilm-involved gene expression. Niosome-encapsulated imipenem’s MBIC was four to six times free imipenem in all the studied isolates, indicating the higher efficiency of nanoparticles compared to free drug. On the other hand, the F1 formulation of Niosome-encapsulated imipenem indicated the highest biofilm formation and growth inhibition. A variety of studies have suggested a direct relationship between the reduced efficiency of antibacterial materials and the ability to form biofilms^49^. Ikonomidis et al. reported that Staphylococcus *MRSE* strains with the ability to form biofilms were more antibacterial resistant than strains unable to form biofilms^50^. Manandhar et al. reported lower sensitivity and therefore higher antibacterial resistance in clinically isolated Staphylococcus strains that could form biofilms compared to those that could not^49^. Formulation F1 of Niosome-encapsulated imipenem indicated the best performance in biofilm growth inhibition, indicating the impact of antibiotic encapsulation on their increased efficiency and biofilm growth inhibition, which is consistent with the results of Barakat et al. Recently, several studies have reported a direct relationship between biofilm gene expression and increased microbial resistance in Staphylococcus *MRSE* strains^40^. Hence, the present study examined the impact of Niosome-encapsulated imipenem sub-MIC concentrations on *icaD*, *FnbA*, *EbpS*, and biofilm genes, revealing a considerable decline in the expression of these genes compared to free imipenem. In addition, the F1 formulation had the highest impact on reducing gene expression and was therefore identified as the optimal formulation. Reducing the expression of the aforementioned genes could result in the inhibition of transcription through a direct impact and cause a reaction between Niosome-encapsulated imipenem and transcription factors, which will result in the inhibition or reduction of the expression of such genes. These results are consistent with those of Lawson et al. (2010), revealing the impact of nanoencapsulation on increasing the inhibition of biofilms and reducing biofilm gene expression^51^. Abdelazizi et al. suggested that Niosome encapsulation of norfloxacin improves its antibiofilm properties and MDR bacterial biofilm formation^52^. The increased microbial resistance to various antibiotics has highlighted the need to find new antibacterial compounds that are nontoxic to mammalian cells^53^. The present study examined the toxicity of various Niosome-encapsulated imipenem formulations to HDFs through the standard method of MTT, indicating a higher cell viability rate in groups treated with Niosome-encapsulated imipenem than in groups treated with free imipenem. Formulation F1 of Niosome-encapsulated imipenem indicated the lowest cytotoxicity compared to other formulations, with a cell viability of over 90% over 24 h. The cytotoxicity of these formulations was due to the use of various Span:Tween surfactant ratios with high biodegradability, making these formulations suitable candidates for encapsulating imipenem as well as other drugs^54^.

## Conclusions

Niosome-encapsulated imipenem is a new approach for restoring imipenem characteristics at a low cost, giving imipenem distinct new properties, such as increased targeted drug delivery, maintaining stability, and controlling drug release. According to this study, this drug formulation is more effective than free imipenem in treating infections due to more resistant *Staphylococcus Epidermidis* isolates that are resistant to a variety of antibiotics, especially vancomycin and methicillin, and the present study is the first to report the impacts of encapsulating imipenem in Niosomes on biofilm formation and growth inhibition, biofilm eradication, and distinct antimicrobial activity against *MRSE* clinical isolates. As a general result, nisosms are promising new drug systems for increasing the antibacterial effects of drugs whose antibacterial features depend on their formulation and composition. According to the present study’s results, it could be inferred that Niosome encapsulation of imipenem increases its antibacterial and antibiofilm activities against MDR and *MRSE S. epidermidis* strains, and these formulations could be considered a new strategy for targeted drug delivery.

## Supplementary Information

Supplementary Figure S1.

## Data Availability

The data that support the findings of this study are available upon reasonable request from the authors.
