# Supplementary material for: Effects of Imipenem-containing Niosome nanoparticles against high prevalence methicillin-resistant Staphylococcus Epidermidis biofilm formed
Source: Sci Rep. 2022 Mar 24;12:5140. doi: 10.1038/s41598-022-09195-9 (PMC8948213; doi:10.1038/s41598-022-09195-9)
Supplement: Supplementary file 1 — Supplementary Figure S1. [file 41598_2022_9195_MOESM1_ESM.docx]

**Supplementary information**

| 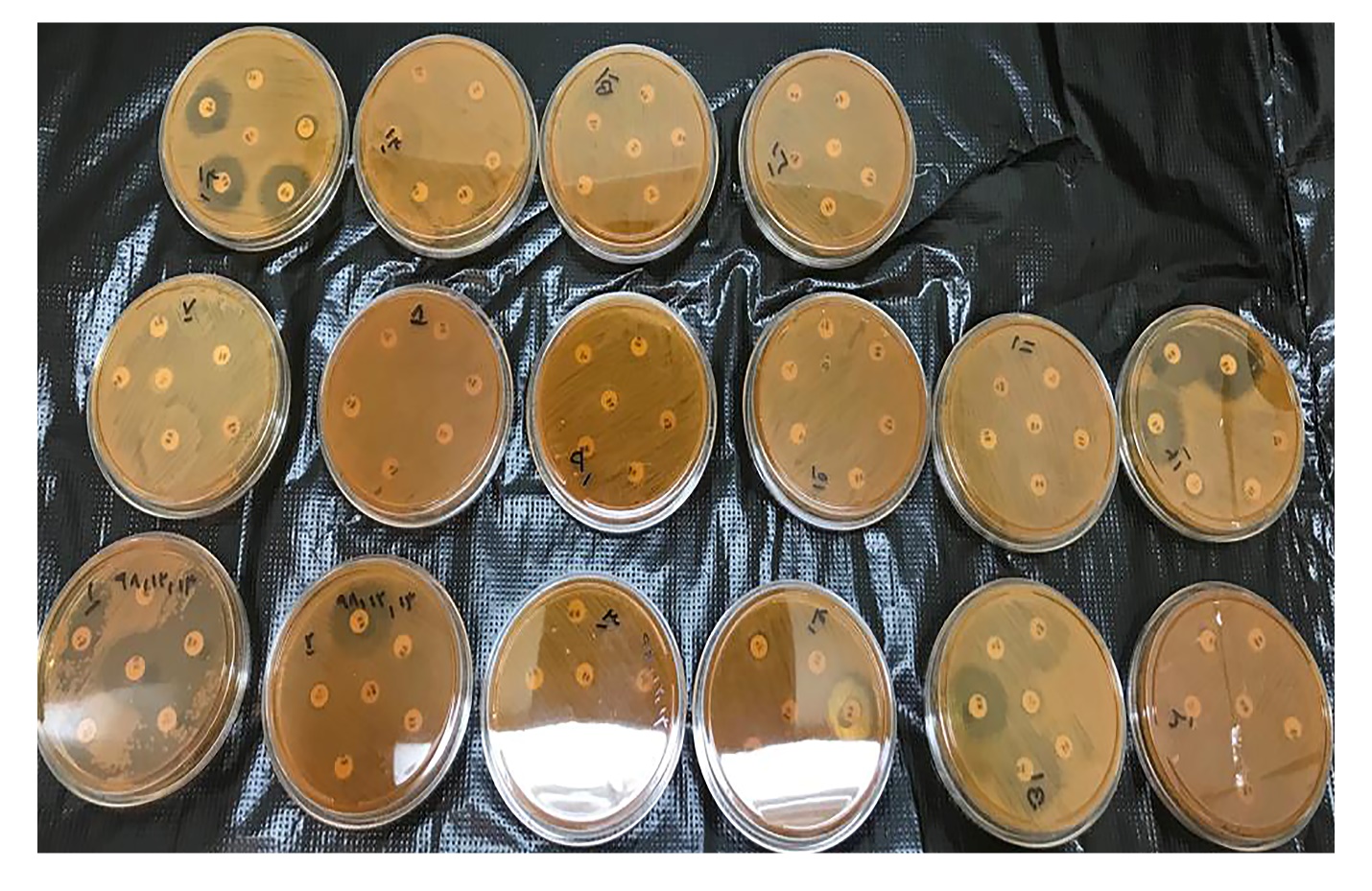 |
| --- |
| Figure S1. Disk diffusion results from some isolates versus selective antibiotics. According to the results, each of the isolates showed a percentage of resistance to different antibiotics. |
